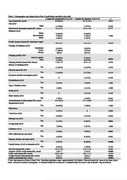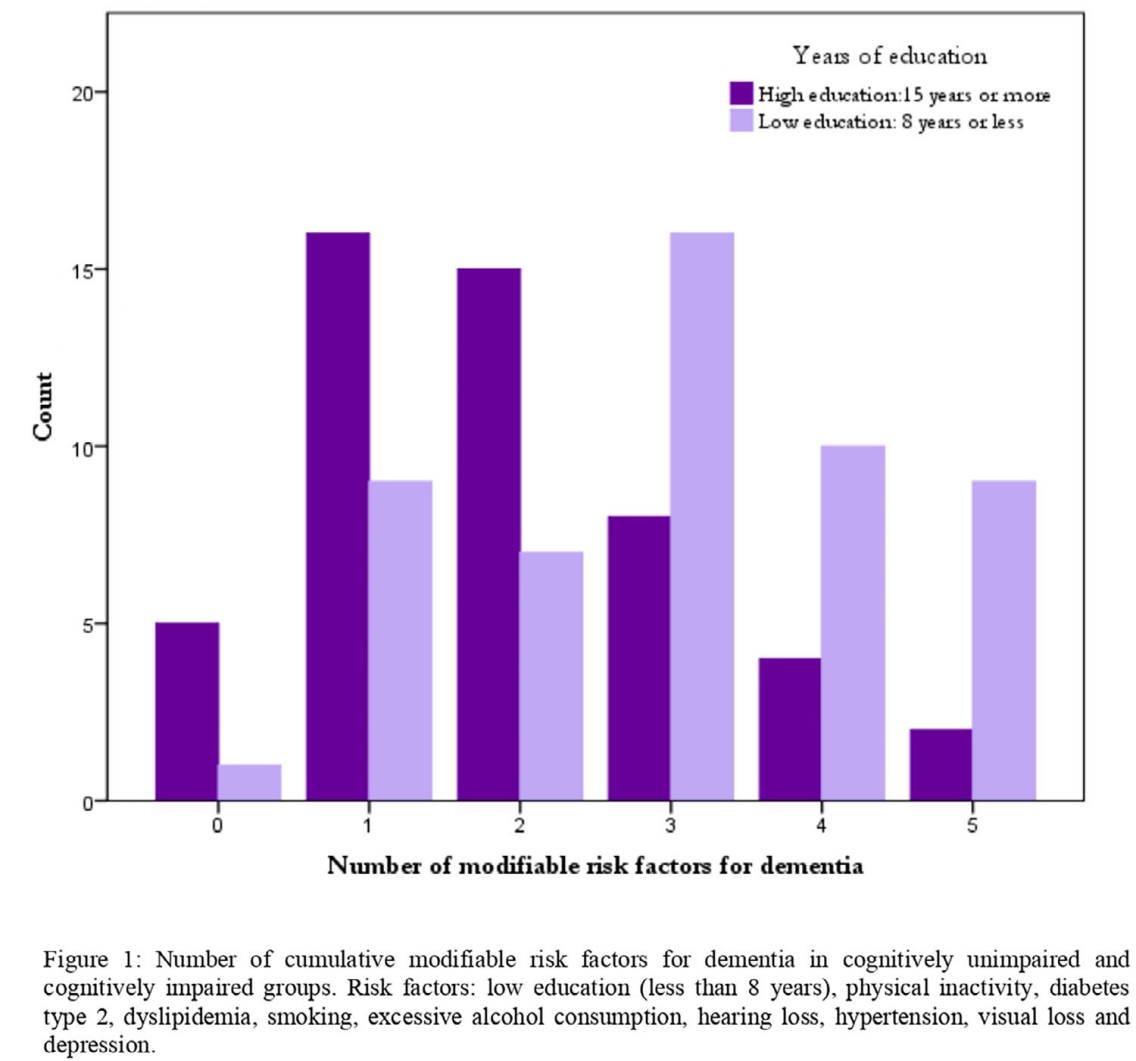# Modifiable risk factors for dementia among individuals of a SUS clinical laboratory in Brazil

**DOI:** 10.1002/alz70860_101737

**Published:** 2025-12-23

**Authors:** Laura Alencastro de Azevedo, Gabrieli dos Santos Battú, Giovanna Carello‐Collar, Christian Limberger, Marco De Bastiani, Lavinia Perquim, Amanda MDL Muliterno Domingues Lourenço de Lima, Simone Martins de Castro, Wyllians Vendramini Borelli, Eduardo R. Zimmer

**Affiliations:** ^1^ Universidade Federal do Rio Grande do Sul, Porto Alegre, Rio Grande do Sul, Brazil; ^2^ Federal University of Health Sciences of Porto Alegre (UFCSPA), Porto Alegre, Rio Grande do Sul, Brazil; ^3^ Universidade Federal do Rio Grande do Sul, Porto Alegre, RS, Brazil; ^4^ Centro de Memória, Hospital Moinhos de Vento, Porto Alegre, RS, Brazil; ^5^ Clinical Hospital of Porto Alegre, Porto Alegre, Rio Grande do Sul, Brazil; ^6^ Brain Institute of Rio Grande do Sul (InsCer), PUCRS, Porto Alegre, Rio Grande do Sul, Brazil; ^7^ McGill Centre for Studies in Agin, Montreal, QC, Canada

## Abstract

**Background:**

Modifiable risk factors for dementia are associated with more than a half of dementia cases. However, most research has been conducted in the Global North, which does not adequately reflect the global diversity and socioeconomic disparities worldwide. Countries in the Global South may exhibit a distinct profile of modifiable dementia risk factors. In this study, we assessed the frequency of dementia risk factors across different cognitive statuses in a Brazilian sample drawn from a SUS (Brazilian health system) clinical laboratory.

**Method:**

We included individuals aged 18 and older who attended a clinical laboratory at a primary care center in Brazil. Cognitive screening was conducted using the Modified Telephone Interview (TICS‐M), and functional independence was assessed with the Lawton Instrumental Activities of Daily Living Scale (IADL). Participants were classified as Cognitively Unimpaired (CU) or Cognitively Impaired (CI) based on cognitive and functional scores, with a cutoff of Quick Dementia Rating System (QDRS) = 2.00 on the QDRS test. Modifiable and non‐modifiable risk factors were evaluated using a structured questionnaire. Statistical analysis was performed using SPSS 20.0 (*p* < 0.05).

**Result:**

We included 43 participants over three months. Twenty‐five (58.1%) were classified as CU and 18 (41.9%) as CI [15 (34.8%) with mild cognitive impairment (MCI) and 3 (7%) with mild dementia]. The CI group reported more frequent feelings of loneliness (*p* = 0.021), poor sleeping quality (*p* = 0.024), hearing loss (*p* = 0.031), imbalance (*p* = 0.041), and memory decline (*p* <0.001, Table 1) compared to the CU group. Also, CI individuals scored higher for anxiety in GAD‐2 (3.3±2.0, *p* <0.001) and for social vulnerability in CSS (4.6±1.4, *p* <0.001) than CU. However, no difference was observed between the groups regarding the cumulative presence of the 10 modifiable risk factors (Figure 1).

**Conclusion:**

The frequency of modifiable and non‐modifiable risk factors for dementia may exhibit a different pattern in Brazil compared to other countries. Further studies are needed to investigate the prevalence of these risk factors to better target vulnerable populations through public health policies.